# Rel Is Required for Morphogenesis of Resting Cells in *Mycobacterium smegmatis*

**DOI:** 10.3389/fmicb.2016.01390

**Published:** 2016-08-31

**Authors:** Mu-Lu Wu, Chuu Ling Chan, Thomas Dick

**Affiliations:** Antibacterial Drug Discovery Laboratory, Department of Microbiology and Immunology, Yong Loo Lin School of Medicine, National University of SingaporeSingapore, Singapore

**Keywords:** stringent response, starvation, bacterial differentiation, SMRC, LARC

## Abstract

Recently we showed that upon transfer of growing *Mycobacterium smegmatis* into saline, the bacilli exited the canonical cell division cycle and formed septated multi-nucleoided cells. Under shock starvation (i.e., in saline without any carbon source), differentiation terminated at this stage with internally remodeled Large Resting Cells (LARCs). Whereas under gentle starvation (i.e., in saline with trace amounts of a carbon source), the septated multi-nucleoided bacilli completed cell division and separated into mono-nucleoided Small Resting Cells (SMRCs). This demonstrated that the non-sporulating mycobacteria are in fact capable of forming morphologically differentiated resting cells when exposed to starvation. Depending on the specific starvation conditions they can form two different resting cell types, LARCs or SMRCs, which share a common cellular differentiation pathway. The mRNA encoding the (p)ppGpp synthetase Rel was found to be transiently upregulated immediately upon starvation under both conditions, suggesting a role for the stringent response factor in both LARC and SMRC development. Here, we disrupted Rel function by generating two types of mutant *M. smegmatis* strains: a *rel* nonsense mutant (*rel*^E4TAG^) in which translation is prematurely terminated at codon 4, and a *rel* deletion mutant (Δ*rel*) in which the entire coding sequence was deleted. Both mutants showed identical phenotypes: sparse septum formation, less DNA compaction, and failure in formation of both the septated multi-nucleoided LARCs and the small-cell morphotype SMRC under starvation conditions. All phenotypes were rescued through the introduction of a wild-type copy of *rel*. Therefore, we conclude that loss-of-function mutations in *rel* block the development of both LARCs and SMRCs by preventing the first morphogenetic step in mycobacterial resting cell development, the formation of septated multi-nucleoided cells. Interestingly, in contrast to Rel’s role in most other bacteria, starvation *survival* was not affected by loss of *rel* function. Our results suggest that Rel may play a starvation-induced morphogenetic role in mycobacteria.

## Introduction

Bacteria are constantly exposed to nutrient limitations in their natural environments (Morita, 1993; Rittershaus et al., 2013). To endure these harsh conditions, most bacteria activate a broadly conserved starvation stress response called the stringent response (Potrykus and Cashel, 2008; Dalebroux and Swanson, 2012; Boutte and Crosson, 2013). Upon nutrient deprivation, the concentration of the signaling nucleotides guanosine tetraphosphate and guanosine pentaphosphate [(p)ppGpp] is rapidly increased by the (p)ppGpp synthetase, RelA/SpoT homologue proteins (RSHs). The elevated cellular concentration of (p)ppGpp alters the transcription of a wide range of genes, thus mediating the physiological adaptation to nutrient starvation and ensuring bacterial survival (Hauryliuk et al., 2015). The stringent response was originally discovered by Stent and Brenner (1961) in *Escherichia coli*. Later it was found to be critical across many bacterial phyla (Braeken et al., 2006; Boutte and Crosson, 2013; Hauryliuk et al., 2015), including mycobacteria (Ojha et al., 2000; Primm et al., 2000; Mathew et al., 2004; Dahl et al., 2005). In mycobacteria, the amount of (p)ppGpp under starvation is mainly modulated by the bifunctional RSH protein Rel which synthesizes and hydrolyzes (p)ppGpp (Avarbock et al., 1999, 2005; Jain et al., 2006). Disruptions of *rel* in mycobacteria were reported to result in defective survival under starvation *in vitro* and loss of virulence in animal infection models (Primm et al., 2000; Dahl et al., 2003, 2005; Klinkenberg et al., 2010).

Mycobacteria are capable of retaining viability under starvation in pure saline for extended periods of time by entering a non-replicating state accompanied by reduced metabolism and increased phenotypic drug resistance, but without any apparent morphological differentiation (Loebel et al., 1933a,b; Nyka, 1974; Betts et al., 2002; Xie et al., 2005; Gengenbacher et al., 2010). Recently, we reported that gentle starvation of *Mycobacterium smegmatis* in saline (PBS) containing traces of a carbon source triggered the development of mono-nucleoided Small Resting Cells (SMRCs), with septated multi-nucleoided cells as intermediates. In contrast, shock starvation in zero-nutrient PBS resulted in apparently unaltered log phase-sized resting cells. Surprisingly, fluorescence microscopic analyses revealed that these shock starved ‘normal sized’ bacilli had remodeled their interior to form septated multi-nucleoided resting cells which we termed Large Resting Cells (LARCs; Wu et al., 2016). Comparative developmental transcriptome analyses showed an early and transient upregulation of the *rel* mRNA under both gentle and shock starvation (Wu et al., under review), suggesting that the mycobacterial (p)ppGpp synthetase may play a role in both LARC and SMRC formation.

In this study, to investigate the function of Rel in nutrient deprivation for *M. smegmatis, rel* mutants were constructed. We showed that loss-of-function mutations in *rel* blocked the development of both LARCs and SMRCs by preventing the formation of septated multi-genomic bacilli, the first cellular differentiation step in mycobacterial resting cell development.

## Materials and Methods

### Bacterial Strains and Culture Conditions

*Mycobacterium smegmatis* mc^2^155 (ATCC 700084) was cultivated at 37°C with agitation in Middlebrook 7H9 broth containing 0.5% bovine albumin, 0.2% glucose, 0.085% NaCl, 0.5% glycerol, 0.0003% catalase, and 0.05% Tween80. In nutrient starvation experiments, mid-log-phase cultures with an optical density at 600 nm (OD_600_) of 0.5 were harvested by centrifugation (3200 rpm, 10 min, 25°C) and washed three times with PBS-0.025% Tween80 or PBS-0.025% Tyloxapol (PBS, Gibco 14080-055). The cultures were then diluted in the corresponding starvation media to a final OD_600_ of 0.10–0.15, and subsequently transferred to a 1-l roller bottle (Corning, COR430195). The starved cultures were incubated for 14 days with rolling at 2 rpm at 37°C. To determine CFUs, appropriate dilutions of cultures were plated on Middlebrook 7H10 agar plates supplemented with 0.5% bovine albumin, 0.2% glucose, 0.085% NaCl, 0.5% glycerol, 0.0003% catalase, and 0.006% oleic acid, and colony numbers were numerated after 2 days incubation.

### Staining and Microscopy

Acid-fast staining was carried out using a TB stain kit (BD, 212520) according to manufacturer’s instructions and observed under a light microscope (Olympus BX60, brightfield). For FM4-64, DNA and Nile red staining, samples were collected at 14 days by centrifugation and then fixed with 4% paraformaldehyde in PBS for 30 min. Fixed samples were then stained and viewed as previously described (Wu et al., 2016). For live/dead staining, LIVE/DEAD BacLight bacterial viability kit (Molecular Probes L7012) was used according to manufacturer’s manual. Briefly, collected live cultures were washed and resuspended in 0.85% NaCl solutions, followed by incubation with equal and proper amount of propidium iodide and Styo 9 in the dark for 15 min. Stained samples were then mounted to slides and observed under epifluorescence microscope Olympus BX60 using the U-MWIG and U-MWIB filters for propidium iodine and Styo, respectively.

### Construction of *rel*^E4TAG^, Δ*rel*, and complemented strains

DNA and mycobacterial genetic manipulations were carried out as described previously (Kaser et al., 2009). The *rel* nonsense mutant *rel*^E4TAG^ was generated by introducing stop codon TAG at the fourth triplet of the *rel* coding sequence (MSMEG_2965) using the point mutation recombineering protocol described by van Kessel and Hatfull (2008). Single-stranded DNA oligos (5′- ACCGCGGGAGGAGACTGCACGGCCTGACCCTTGCCTGGCTAGTCGACCATGGTTGTCACCTCCTGCCCAGCAACCCGAATT-3′) harboring the desired point mutation at the center were introduced into electrocompetent *M. smegmatis* containing pJV62. Positive mutants were screened using PCR amplification (5′- GTGACAACCATGGTCGACT-3′ and 5′-TTCGGGTAGATCTCGCGGT-3′) and verified by DNA sequencing (5′- CCGAAGGAATTGACATCGC-3′, and 5′-TTCGGGTAGATCTCGCGGT-3′; Supplementary Figure 1A). Deletion of the *rel* coding sequence was carried out by gene replacement recombineering method as described in van Kessel et al. (2008). A 500 bp fragment upstream of *rel* was amplified using primers 5′-ACGTCTTAAGCCAGTTCAAGGACCTCACGCC-3′ and 5′- CTAGTCTAGAGGTTGTCACCTCCTGCCCAG-3′. Similarly, a 513 bp fragment downstream of *rel* (including the last six nt in *rel* coding sequence) was amplified using 5′- CTCGAAGCTTGCCTGATCGAGCGGTTCGGCGT-3′ and 5′- CTAGACTAGTCTCCTCGTGGACAGGATCGGCCGA-3′. These two fragments were sequentially cloned into vector pYUB854 flanking the hygromycin resistance cassette at *Afl*II*/Xba*I and *Hind*III*/Spe*I sites, respectively, to generate pYUB854-*rel*KO. 2.9 kb double-stranded allelic exchange substrates obtained by digesting pYUB854-*rel*KO with *Afl*II and *Spe*I-HF were electroporated into *M. smegmatis* containing vector pJV53 to generate *rel* knockout strain Δ*rel*. The complete *rel* coding sequence (except the last six nt) was verified to be replaced by the hygromycin^R^ cassette via PCR analysis (Supplementary Figures 1B,C) and DNA sequencing. For complementation, the *rel* coding sequence from *Mycobacterium tuberculosis* (Rv2583c) including the 260 bp upstream containing the native *rel* promoter was isolated via PCR amplification with the primer set 5′- CTCGAAGCTTACGGCGCCGCCACTCTGGAGATTC-3′ and 5′- ACGTGGTACCCTACGCGGCCGAGGTCACCCGGTA-3′, and cloned into the *Hind*III and *Kpn*I sites of the integrative mycobacterial shuttle vector pMV306 to generate plasmid pMV306-Rel_Mtb_. The *rel_Mtb_* sequence in the complementation construct pMV306-*rel_Mtb_* was verified by DNA sequencing. Successful integration of *M. tuberculosis rel* allele into the Δ*rel* background was verified by positive PCR amplification with primer set 5′-ACAACATGCGCACCATGCG-3′ and 5′-CAAGCGCTGCAACGGAAGTC-3′ (Supplementary Figure 1C, lane 3). Due to the high homology of *rel* alleles between *M. tuberculosis* and *M. smegmatis*, this set of primers binds to both wild-type *M. smegmatis* DNA and the Δ*rel* complemented strain (Δ*rel*Comp) that contains the *M. tuberculosis rel* copy.

## Results

### Rel-Disrupted *M. smegmatis* Mutants Fail to Form SMRCs under Gentle Starvation

**FIGURE 1:**
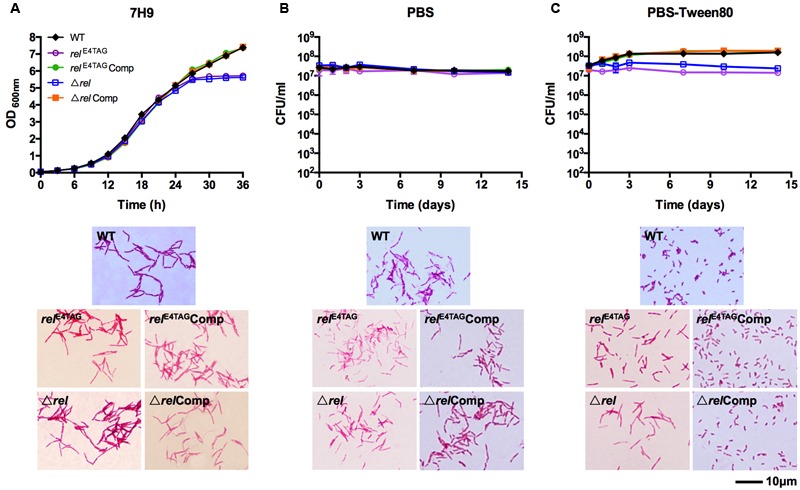
**Growth, starvation survival, and cell size phenotypes of *Mycobacterium smegmatis rel*^E4TAG^ and Δ*rel*. (A)** Growth curves and acid-fast stained log-phase culture samples of *M. smegmatis rel*^E4TAG^ and Δ*rel* in nutrient broth in comparison with wild-type *M. smegmatis* and the two complemented mutant strains (*rel*^E4TAG^Comp and Δ*rel*Comp). **(B,C)** Survival curves and acid-fast stained 14-day starved samples of *M. smegmatis rel*^E4TAG^ and Δ*rel* in **(B)** PBS and **(C)** PBS-Tween80 in comparison with wild-type *M. smegmatis* and the two complemented strains. In all curves, black diamond: wild-type strain; purple empty circle: *rel*^E4TAG^ nonsense mutant; green filled circle: *rel*^E4TAG^Comp; blue empty square: Δ*rel* knockout strain; orange filled square: Δ*rel*Comp. Growth curves and survival curves data are shown as means ± standard deviations from two independent biological replicates. Representative fields were chosen for acid-fast stained images.

To determine the role of Rel in mycobacterial resting cell differentiation, we introduced a TAG stop codon at the fourth triplet of the *rel* coding sequence via oligonucleotide-based site directed genome mutagenesis (‘recombineering’) to block synthesis of the protein (see Material and Methods for details). The verified *rel* nonsense mutant, named *rel*^E4TAG^, was then subjected to growth in nutrient broth, shock starvation in PBS, or mild starvation in PBS containing traces of Tween80, a fatty acid ester used by mycobacteria as a carbon source (Tomioka, 1983). **Figure 1A** (*rel*^E4TAG^) shows that the *rel* nonsense mutation did not affect the growth rate or cell shape of *M. smegmatis* in nutrient broth (Gupta et al., 2016), but the mutant strain entered into stationary phase earlier than wild-type bacilli. Surprisingly, the *rel* nonsense mutation did not affect survival of the bacilli when subjected to starvation conditions (**Figures 1B,C**). Microscopic analyses of acid-fast stained samples depicted in **Figure 1C** showed that the *M. smegmatis rel* nonsense mutant grown under gentle starvation conditions (PBS-Tween80) failed to form SMRCs. Only 8% of cells in starved *rel* nonsense mutant cultures were of the SMRC morphotype, whereas 95% of cells in wild-type cultures were SMRCs (*n* = 100), suggesting a role of Rel in the formation of the small-cell morphotype. Consistent with this microscopic observation, the slight increase in CFU observed for wild-type bacteria under gentle starvation due the separation of the septated multi-nucleoided cell intermediates into mono-nucleoided SMRCs, was not observed for *M. smegmatis rel*^E4TAG^ (**Figure 1C**). Both the microscopic and the CFU phenotype could be restored by introduction of a wild-type allele of *M. tuberculosis rel* under its native promoter into the *M. smegmatis rel* nonsense mutant background (**Figure 1C**, *rel*^E4TAG^Comp). No apparent cell size difference was observed in acid-fast stained *rel* nonsense mutants in PBS starved cultures (**Figure 1B**, *rel*^E4TAG^). Taken together, analyses of a *rel* nonsense mutant suggest that Rel is required for the formation of SMRCs.

To exclude the possibility that the *rel* nonsense mutant may be leaky, i.e., allows some translational read-through of the stop codon, we generated a *M. smegmatis* Δ*rel* strain in which the entire coding sequence of *rel* was deleted and replaced with a hygromycin resistance cassette (see Material and Methods for details). **Figure 1** (Δ*rel*) shows that all the phenotypes displayed by the Δ*rel* loss-of-function mutant were identical to that observed for the *rel* nonsense mutant, demonstrating that the introduced stop codon in the nonsense mutant effectively blocked expression of the Rel product. As shown for the *rel* nonsense mutant, deletion of *rel* blocked the formation of SMRCs (only 5% of Δ*rel* mutants formed small cells, *n* = 100) and this phenotype was rescued by complementation with the wild-type allele of the gene(**Figure 1C**, Δ*rel*Comp).

### *rel* Loss-of-Function Mutation Blocks the First Step in Mycobacterial Resting Cell Formation – The Generation of Septated Multi-nucleoided Cells

**FIGURE 2:**
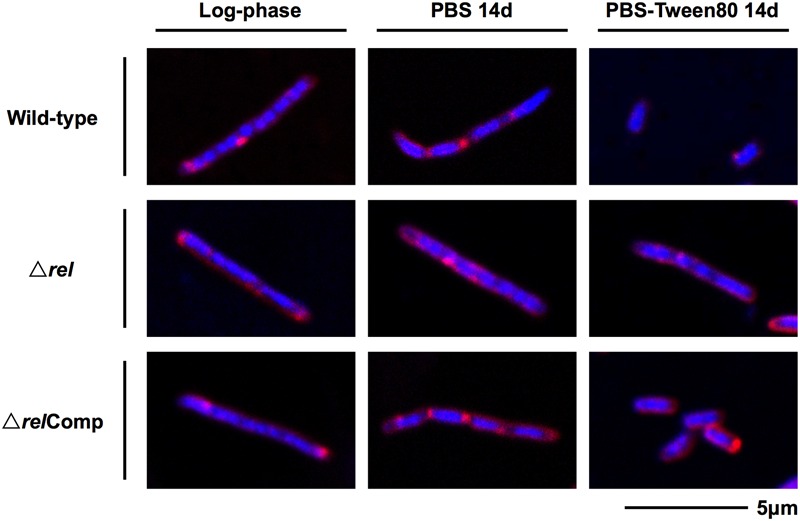
**DNA and membrane staining images of *M. smegmatis* Δ*rel* in comparison with wild-type and complemented strain.** Fluorescent probe stained culture samples of *M. smegmatis* Δ*rel* grown in nutrient broth (log-phase) and starved in PBS or PBS-Tween80 for 14 days in comparison with wild-type *M. smegmatis* and *M. smegmatis* Δ*rel* complemented strain (Δ*rel*Comp) are shown. Staining was done with DAPI (blue) to visualize DNA and FM4-64 (red) to visualize membranes. Representative fields were shown.

**FIGURE 3:**
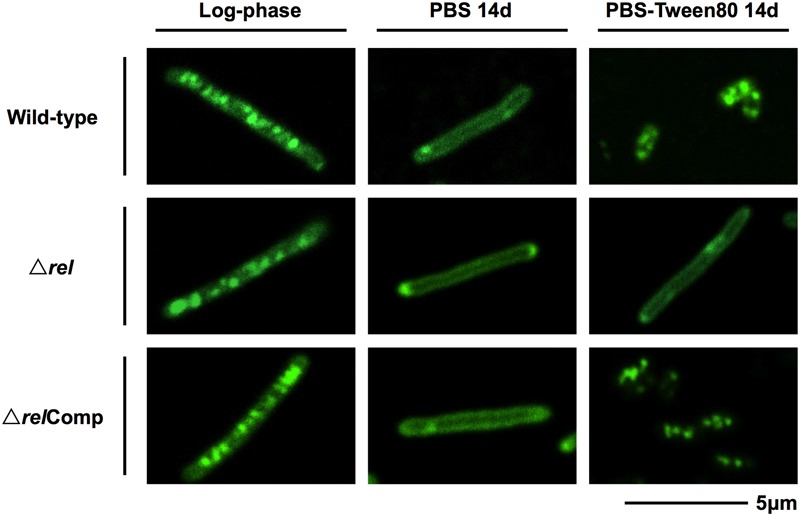
**Lipid body staining images of *M. smegmatis* Δ*rel* in comparison with the wild-type and complemented strain.** Fluorescent probe stained culture samples of *M. smegmatis* Δ*rel* grown in rich broth (log-phase) and starved in PBS or PBS-Tween80 for 14 days in comparison with wild-type *M. smegmatis* and *M. smegmatis* Δ*rel* complemented strain (Δ*rel*Comp) are shown. Staining was done with Nile red (green) to visualize intracellular lipid bodies. Nile red stains predominantly intracellular lipid inclusions. Representative fields were shown.

The acid-fast staining results showed that *M. smegmatis rel* loss-of-function strains failed to form SMRCs in gently starved cultures (**Figure 1C**). To determine whether loss of *rel* function affects the formation of the LARC-like septated, multi-nucleoided intermediates during SMRC development and/or the formation of LARCs under conditions of shock starvation, we carried out fluorescence microscopic analyses on *M. smegmatis* Δ*rel* cultures subjected to gentle PBS-Tween80 and shock PBS starvation. Starved cultures were stained with the fluorescent probes DAPI and FM4-64 to visualize DNA and membrane structure, respectively. As reported in our previous study (Wu et al., 2016), around 70% of wild-type *M. smegmatis* cells formed septated multi-nucleoided bacilli (containing 1–3 septa per cell) under shock starvation. In contrast, **Figure 2** shows that Δ*rel* bacilli failed to form septated cells in PBS. Only sparse septum formation (none or one) was observed in shock-starved Δ*rel* bacilli: 72% of the cells did not contain any septum and 26% showed one septum (*n* = 70). Likewise, in PBS-Tween80 cultures where the majority of the wild-type cells produced SMRCs via a septated multi-nucleoided intermediate stage (Wu et al., 2016), few septated multi-nucleoided cells were observed: 66% of Δ*rel* bacilli did not contain any septum while 27% had only one septum (*n* = 100). In addition to the septation pattern, DNA segregation and compaction were also affected by the absence of Rel protein. In contrast to the highly condensed nucleoids observed in starved wild-type cells, DNA in Δ*rel* bacilli distributed throughout the whole cell under both starvation conditions, i.e., the DNA was less condensed (**Figure 2**). Furthermore, loss of *rel* function resulted in the loss of intracellular lipid bodies in PBS-Tween80 starved cultures (**Figure 3**). Unexpectedly, we observed additional cell structures in PBS shock-starved *rel* mutant cultures upon DAPI and Nile red staining and these structures will be discussed below. Analyses of the *M. smegmatis* loss-of-function strain complemented with the wild-type allele of *rel* restored septum formation, DNA compaction and intracellular lipid body accumulation phenotypes (**Figures 2** and **3**, Δ*rel*Comp). These results indicate that Rel is required for both LARC and SMRC formation and that loss-of-function of *rel* blocks the first cellular step of mycobacterial resting cell formation – the generation of septated multi-nucleoided cells. This genetic function of *rel* is consistent with the observation that the *rel* gene is transiently upregulated under both culture conditions prior to the onset of septation and nucleoid compaction (Wu et al., under review).

**FIGURE 4:**
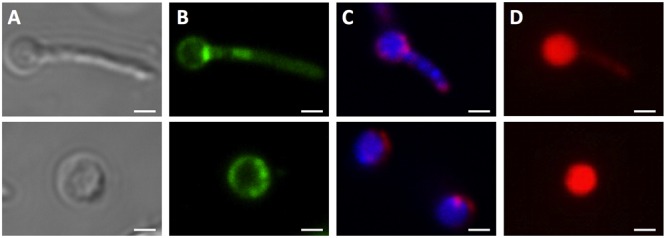
**Unusual cell shapes in PBS-starved cultures of *M. smegmatis* Δ*rel*.** Typical examples of unusual ‘ball-like’ cell shapes (∼60%) observed in 14-day PBS-starved *M. smegmatis* Δ*rel* cultures are shown. Cells carried ball-like appendices are shown in the upper panels, and ball-shaped cells are shown in the lower panels. **(A)** Differential interference contrast images to visualize cell shapes. **(B)** Nile red (green) stained samples to visualize lipid bodies. **(C)** DAPI (blue) and FM4-64 (red) double stained samples to visualize DNA and membrane, respectively. **(D)** Propidium iodide (red) staining was done to show breakdown of the permeability barrier of the ball-like structures. These structures were observed under phase contrast but not in acid-fast stained samples, suggesting that these structures may be cell wall defective. Representative fields were shown. White bar = 1 μm.

It is interesting to note that during fluorescence microscopic analyses using DAPI or Nile red stain, additional cell morphotypes were observed specifically in 14-day old PBS shock starved *M. smegmatis* Δ*rel* cultures. These novel cell structures, either containing a ball-like attachment (**Figure 4**, **upper row**) or being ball-like (**Figure 4**, **lower row**), were comparable in number to the number of ‘intact’ acid-fast positive bacilli described above under these culture conditions. Notably, these ball-like structures were not observable in acid-fast stained samples, suggesting that they have an altered cell wall thus affecting the staining behavior. These structures were also only weakly stained by FM4-64. Moreover, these structures were permeable to propidium iodide (**Figure 4D**), indicating a breakdown of the cell permeability barrier. It remains to be determined whether these DNA-containing structures are dead or injured cells with compromised cell permeability, as propidium iodide stained cells do not necessarily represent dead cells (Shi et al., 2007).

## Discussion

**FIGURE 5:**
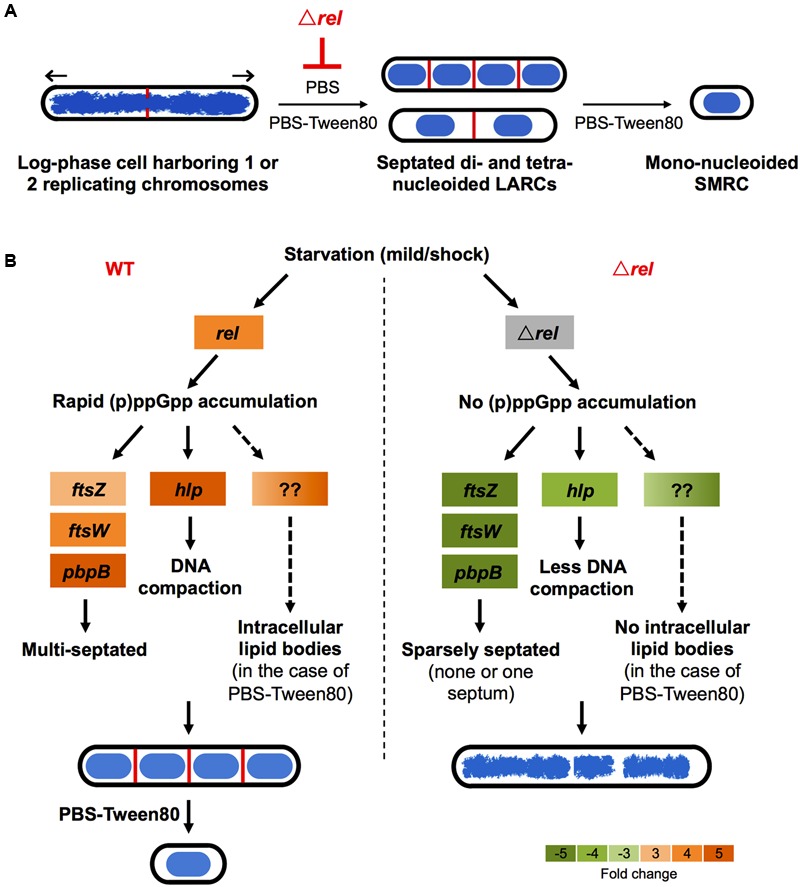
**Schematic of Rel’s potential roles in SMRC (*Sm*all *R*esting *C*ell)/LARC (*La*rge *R*esting *C*ell) development. (A)** Model depicting the nutrient starvation induced cellular differentiation program in *M. smegmatis*. Loss of *rel* function blocks the formation of septated multi-nucleoided cells seen as LARCs or the intermediates in SMRC formation. Blue: DNA, red: septa, black: cell envelope. Arrows indicate polar growth of the replicating log-phase bacilli. **(B)** Model on how Rel may be involved in the formation of the first cellular differentiation step, the formation of septated, multi-genomic cells with condensed nucleoids. For the left panel, expression changes are shown as fold changes of 3 h-starved cultures compared with log-phase cells as described in Wu et al. (under review). Orange colors indicate increase in transcript levels. For the right panel, expression changes of *ftsW, pbpB*, and *hlp* are shown as fold changes of 6 h-starved Δ*rel* mutant vs. 6 h-starved wild-type *Mycobacterium tuberculosis* as reported by Dahl et al. (2003), while the transcript level of *ftsZ* is shown as fold change of starved Δ*rel* mutant vs. starved wild type *M. smegmatis* as reported by Gupta et al. (2016). Green colors indicate decrease in transcript levels.

Recently, we showed that “non-sporulating” *M. smegmatis* is in fact capable of forming morphologically distinct resting cells. Gentle starvation in PBS with traces of a carbon source triggers the development of mono-nucleoided SMRCs, with septated multi-nucleoided cells as intermediates. Shock starvation in PBS with zero carbon source results in the formation of LARCs, which are similar in shape and size to log phase cells. However, compared to growing bacilli, LARCs show *internal* remodeling to form septated multi-nucleoided cells, similar to the intermediates observed during SMRC development. Based on these observations, we proposed that *M. smegmatis* harbors a novel starvation-induced morphological differentiation program: Upon nutrient limitation, bacilli exit the canonical cell division cycle and form septated multi-nucleoided cells. Under zero-nutrient conditions, the bacilli terminate development at this stage as LARCs. But in the presence of traces of a carbon source, these multi-nucleoided cells continue differentiation. They complete cell division and separate into mono-nucleoided SMRCs (**Figure 5A**; Wu et al., 2016). To begin to dissect the genetic program underlying SMRC/LARC differentiation, we generated loss-of-function mutations in one of the early and transiently upregulated genes common to SMRCs and LARCs, the stringent response factor *rel*. Fluorescence microscopic analyses showed that the *rel* mutations prevented the first cellular differentiation step in SMRC/LARC formation – the generation of septated multi-genomic cells with condensed chromosomes (**Figure 5A**). Interestingly, starvation *survival* of the bacilli was not affected in *rel* loss-of-function strains, suggesting a role for the mycobacterial Rel in starvation-induced morphogenesis rather than in starvation survival.

### Stringent Response vs. Starvation Survival

The lack of a starvation survival phenotype for our loss-of-function mutants was surprising because it was previously reported that a *M. smegmatis rel* mutant showed ∼2-log reduction of CFU after 28-day starvation in Tris-buffered saline-Tween80 (TBST; Dahl et al., 2005). However, we did not observe any loss of viability in our *M. smegmatis* Δ*rel* mutant. To investigate this difference, the nutrient starvation experiments were repeated in TBST, but we observed the same results as with PBS whereby there was no loss of viability (data not shown). The reason for the discrepancy remains to be determined. However, it was noted that the authors only deleted part of the coding sequence, the (p)ppGpp synthetase domain, leaving the (p)ppGpp hydrolase domain of Rel intact (Dahl et al., 2005).

Recently, in various bacterial species, small alarmone synthetases (SASs) – small proteins which contain only the synthetase domain – have been discovered as alternative contributors to the cellular (p)ppGpp level (Hauryliuk et al., 2015). In 2012, Murdeshwar and Chatterji (2012) reported the presence of a second monofunctional (p)ppGpp synthetase in the *M. smegmatis* genome. This short alarmone synthetase, named MS_RHII-RSD, was reported to constitutively produce a basal level of (p)ppGpp in the exponential growth phase, as well as under starvation and other stress conditions, including oxidative stress, osmotic stress, and acidic stress (Murdeshwar and Chatterji, 2012). An alternative source of (p)ppGpp provided by such SASs has been shown to contribute to bacterial metabolic balance and stress tolerance in other bacterial species, e.g., *Enterococcus faecalis, Vibrio cholera*, and *Bacillus subtilis* (Nanamiya et al., 2008; Das et al., 2009; Gaca et al., 2013). It has also been shown that small amounts of (p)ppGpp produced in a leaky, Tet-controlled hydrolase-dead *rel* in *M. tuberculosis* was sufficient to restore the survival phenotype of Δ*rel* mutants in a mouse model (Weiss and Stallings, 2013). Although the bifunctional Rel is the principal enzyme responsible for the rapid accumulation of (p)ppGpp upon nutrient starvation, the basal level of (p)ppGpp provided by MS_RHII-RSD may be sufficient for survival of *M. smegmatis* Δ*rel* mutant under nutrient starvation in the absence of Rel. The intact hydrolase domain left in the *rel* synthetase-dead mutant constructed by Dahl et al. (2005) might have reduced the basal (p)ppGpp level generated by MS_RHII-RSD, resulting in the reported loss of viability under starvation. In addition, it is interesting to note that deletion of the (p)ppGpp hydrolase domain alone has been shown to be lethal in *E. coli, V. cholera*, and *M. tuberculosis* in the presence of a functional (p)ppGpp synthetase (An et al., 1979; Nanamiya et al., 2008; Das et al., 2009; Weiss and Stallings, 2013). The fact that deletion of Rel which contains the only known hydrolase in *M. smegmatis* is not fatal suggests that there might exist an alternative pathway of (p)ppGpp degradation in *M. smegmatis*.

### Stringent Response vs. Morphological Differentiation

The involvement of the stringent response in morphological differentiation, mediated by a bifunctional Rel, has been reported for various differentiating bacterial species. Loss of *rel* blocks the initiation of the endosporulation process in *B. subtilis* as well as fruiting body development in *Myxococcus xanthus* (Ochi et al., 1981; Harris et al., 1998). In *Streptomyces coelicolor*, which belongs to the same Actinobacteria phylum as mycobacteria, deletion of *rel* did not affect vegetative growth but formation of exospores (Hesketh et al., 2007). The exact mechanism of how Rel controls the early SMRC/LARC developmental process remains to be determined. In *Streptomyces*, the (p)ppGpp-mediated effect on differentiation is thought to work via reduction of the cellular GTP concentration. In *Streptomyces rel* loss-of-function strains, the GTP level is not reduced and differentiation is blocked (Ochi, 1987). To determine whether GTP depletion may play a role for mycobacterial differentiation, we attempted to prevent GTP depletion by adding guanosine or GTP (up to 2 mM) to gently starved *M. smegmatis* cultures. However, the bacilli still formed SMRCs. The complementary experiment trying to restore the SMRC formation in starved *M. smegmatis* Δ*rel* by lowering the GTP level was also not conclusive: Exposure of starved *M. smegmatis* Δ*rel* cultures to decoyinine, a GMP synthetase inhibitor (Mitani et al., 1977), to block GTP biosynthesis and thus lower GTP levels, did not generate SMRCs (data not shown). However, we cannot rule out the potential role of GTP in the (p)ppGpp-mediated differentiation process as guanosine and GTP might not penetrate the mycobacterial cell envelope and decoyinine might not be able to inhibit mycobacterial GMP synthetase.

Intriguingly, some key genes found to be upregulated during development of SMRCs and LARCs (**Figure 5B**, left panel; Wu et al., under review) appear to be under either direct or indirect control of Rel as summarized in **Figure 5B** (right panel). The transcript level of *ftsZ*, which encodes the septum formation protein, has been shown to be significantly down-regulated in *rel*-disrupted strains of *M. smegmatis* (Gupta et al., 2016). Likewise, the expression of two other septum-formation components FtsW and PBPB was four–fivefolds less in a *M. tuberculosis rel* deletion strain compared to the wild-type strain under starvation (Dahl et al., 2003). Moreover, the DNA condensation protein Hlp, showed a decreased transcript level in a *M. tuberculosis rel* deletion strain (Dahl et al., 2003). Reduced expression of these genes in the absence of Rel might explain the failure of SMRC/LARC formation, including the absence of nucleoid compaction observed in our Δ*rel* mutant. Another appealing phenotype observed in our *M. smegmatis* Δ*rel* mutant is the absence of intracellular lipid bodies (proposed to be composed of triacylglycerol; Garton et al., 2002; Daniel et al., 2004) in gently starved bacilli. Although no key genes involved in triacylglycerol biosynthesis and hydrolysis (e.g., *tgs1* and genes encoding lipases) and downstream fatty acid utilization (e.g., *aceA*) have been shown to be under direct regulation of Rel in mycobacteria, (p)ppGpp has been suggested to regulate lipid metabolism by inhibiting fatty acid biosynthesis (Heath et al., 1994), but how the stringent response factor mediates the lipid body persistence in SMRCs remains to be determined.

## Conclusion

Our *rel* knockout studies showed that the mycobacterial stringent response factor is required for the formation of both SMRCs and LARCs. Loss of *rel* function blocks the first cellular differentiation step in the formation of mycobacterial resting cells – the formation of the septated multi-nucleoided cells. Rel is not required for maintaining viability of starved cells. Collectively, this suggests a morphogenetic rather than a survival function for the stringent response factor in *M. smegmatis*. A low basal level of (p)ppGpp (perhaps produced by MS_RHII-RSD) may be sufficient for survival under starvation, while a high level of (p)ppGpp (requiring functional Rel) may be necessary for morphological differentiation.

## Author Contributions

M-LW and TD conceived the project and designed the strategy. CC generated the *relA* nonsense mutant and carried out the preliminary studies. M-LW carried out all the other experiments. M-LW and TD analyzed the data and wrote the manuscript.

## Conflict of Interest Statement

The authors declare that the research was conducted in the absence of any commercial or financial relationships that could be construed as a potential conflict of interest.
