# Supplementary material for: Rel Is Required for Morphogenesis of Resting Cells in Mycobacterium smegmatis
Source: Front Microbiol. 2016 Aug 31;7:1390. doi: 10.3389/fmicb.2016.01390 (PMC5005932; doi:10.3389/fmicb.2016.01390)
Supplement: Supplementary file 1 [file Presentation_1.PDF]

Supplementary Material

Rel is required for morphogenesis of resting cells in *Mycobacterium smegmatis*

Mu-Lu Wu, Chuu Ling Chan, Thomas Dick*

***Correspondence:** Thomas Dick: thomas_dick@nuhs.edu.sg

1 Supplementary Figure

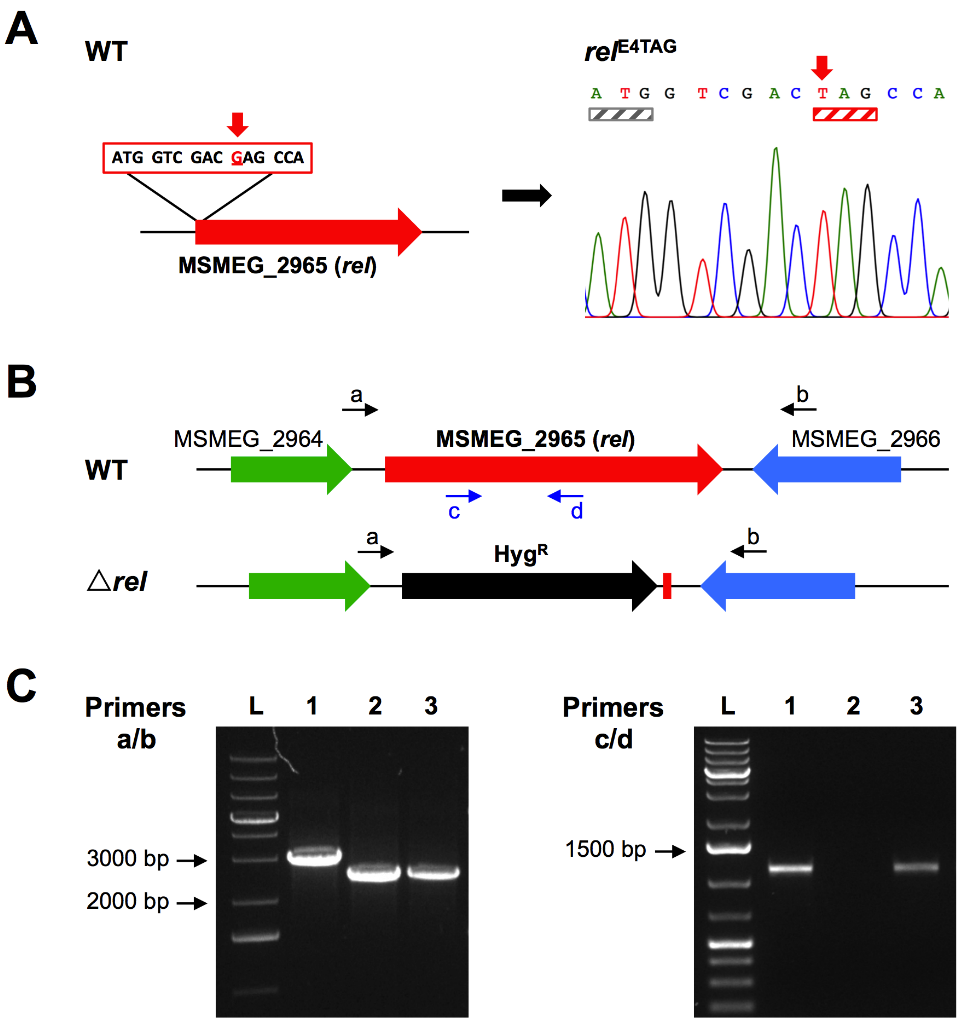

Supplementary Figure 1. Construction of *M. smegmatis rel* nonsense mutant strain *rel*^{E4TAG} and *rel* deletion strain △*rel*. (A) The 5' region of the coding sequence of *rel* in wild-type *M. smegmatis* and the *rel* nonsense mutant are shown. The *rel* nonsense mutant *rel*^{E4TAG} was generated by introducing a point mutation at the 10th nucleotide (G$\rightarrow$T) of the *rel* coding sequence to change the glutamic acid codon into the stop codon TAG. Successful mutation was confirmed by DNA sequencing. The grey box with stripes indicates the start codon while the red box with stripes indicates the newly introduced stop codon. **(B)** Gene loci of *rel* wild type and deletion mutant are shown. The *rel* deletion strain △*rel* was constructed by replacing the complete *rel* coding sequence (except the last 6 bp) with a hygromycin resistance cassette. **(C)** Agarose gel electrophoretic analyses of the PCR products generated by primers a/b and c/d depicted in (B) are shown. L, DNA ladder; 1, wild-type *M. smegmatis*; 2, △*rel* strain; 3, △*rel*Comp strain. The product amplified by primers a/b with △*rel* DNA is 467 bp smaller than that obtained from the wild-type DNA, confirming the successful replacement of the wild-type *rel* allele by the hygromycin resistance cassette. The complemented strain (△*rel*Comp) was generated by introducing a wild-type *M. tuberculosis rel* copy under control of its native promoter into the △*rel* mutant background. Due to the high homology of *rel* between *M. tuberculosis* and *M. smegmatis*, *rel* internal primers c/d produce PCR products with both wild-type *M. smegmatis rel* DNA and DNA extracted from *M. smegmatis* △*rel*Comp. Deletion of the complete *rel* sequence in △*rel* was also verified by DNA sequencing of the PCR products.
